# High cortisol levels are associated with oxidative stress and mortality in maintenance hemodialysis patients

**DOI:** 10.1186/s12882-022-02722-w

**Published:** 2022-03-08

**Authors:** Juhee Kim, Kyu-sang Yun, Ajin Cho, Do Hyoung Kim, Young-Ki Lee, Myung-Jin Choi, Seok-hyung Kim, Hyunsuk Kim, Jong-Woo Yoon, Hayne C. Park

**Affiliations:** 1 Department of Internal Medicine, Kangnam Sacred Heart Hospital, Hallym University College of Medicine, 1, Singil-ro, Yeongdeungpo-gu, Seoul, 07441 Republic of Korea; 2 Hallym University Kidney Research Institute, Seoul, Korea; 3 Department of Internal Medicine, Korean Armed Forces Capital Hospital, Seongnam, Korea; 4 Department of Internal Medicine, Chuncheon Sacred Heart Hospital, Hallym University College of Medicine, Chuncheon, Korea

**Keywords:** Aldosterone, Cortisol, Heart Failure, Systolic, Mortality, Oxidative Stress

## Abstract

**Background:**

Chronic stimulation of the mineralocorticoid receptor has been suggested as one of the potential causes of cardiovascular events and death in patients with end-stage renal disease. This observational cohort study was performed to demonstrate that serum cortisol might be a predictive marker for patient mortality and to evaluate its association with oxidized low-density lipoprotein (oxLDL) in hemodialysis (HD) patients.

**Methods:**

Patients receiving HD three times a week were screened for enrollment at two institutions. Baseline cortisol levels were measured before each HD session, and the patients were divided into two groups according to the median value of serum cortisol before analysis. The baseline characteristics and laboratory values of the high and low cortisol groups were compared. Serum cortisol, adrenocorticotropic hormone, renin, aldosterone, and oxLDL were measured in 52 patients to evaluate the effect of oxidative stress on serum cortisol levels.

**Results:**

A total of 133 HD patients were enrolled in this cohort study. Compared to the patients with low serum cortisol levels, the patients with high serum cortisol levels (baseline cortisol ≥ 10 μg/dL) showed higher rates of cardiovascular disease (59.7% vs. 39.4%, *P*=0.019) and left ventricular systolic dysfunction (LVSD) (25.9% vs. 8.0%, *P*=0.016). The patients in the high cortisol group demonstrated higher all-cause mortality than those in the low cortisol group. The serum cortisol level was an independent risk factor for patient mortality (hazard ratio 1.234, 95% confidence interval 1.022-1.49, *P*=0.029). Among the 52 patients with oxLDL measurements, oxLDL was an independent risk factor for elevated serum cortisol levels (Exp(B) 1.114, *P*=0.013) and LVSD (Exp(B) 12.308, *P*=0.045). However, plasma aldosterone levels did not affect serum cortisol levels.

**Conclusions:**

Serum cortisol is a useful predictive marker for all-cause death among patients receiving HD. OxLDL is an independent marker for elevated serum cortisol among HD patients.

**Supplementary Information:**

The online version contains supplementary material available at 10.1186/s12882-022-02722-w.

## Background

Patients with end-stage renal disease demonstrate higher cardiovascular morbidity and mortality than those with normal renal function or mild renal failure [1]. The chronic stimulation of mineralocorticoid receptors by either aldosterone or cortisol has been suggested as a possible cause of sudden cardiac death in diabetic patients receiving hemodialysis (HD) [2]. Aldosterone is a main effector hormone that contributes to deleterious cardiac damage [3–5]. However, there has been controversy about the harmful effect of plasma aldosterone on cardiovascular outcomes among HD patients. Some observational studies with a small number of HD patients showed a positive association [6, 7] while others reported a negative association [8–10] with cardiovascular outcome and mortality.

Serum cortisol is another hormone that can bind to the mineralocorticoid receptor to produce adverse cardiovascular outcomes in HD patients. Under normal conditions, cortisol is converted into inactive cortisone by the 11β hydroxysteroid dehydrogenase type 2 (11βHSD2) enzyme. However, the activity of 11βHSD2 decreases as renal function declines, and therefore, cortisol levels increase disproportionately compared to aldosterone levels [11]. Since 11βHSD2 is minimally expressed in the heart, cardiac mineralocorticoid receptors may be mainly activated by cortisol rather than aldosterone in HD patients. Indeed, the serum cortisol level was reported to be one hundred to one thousand times higher than the plasma aldosterone level in such patients [12]. In addition, previous studies have shown the harmful effect of serum cortisol in HD patients: HD patients with high serum cortisol levels had high rates of cardiovascular events and mortality [2, 13, 14].

Meanwhile, low-density lipoprotein (LDL) is a major source of cholesterol for aldosterone and cortisol synthesis. Under circumstances of oxidative stress, LDL may be modified to oxidized LDL (oxLDL), which is highly atherogenic. The concentration of oxLDL is elevated in patients with chronic kidney disease in part because of the inability of high-density lipoprotein to reduce oxLDL and in part because of the increased LDL lifespan due to decreased renal clearance [15]. A previous study demonstrated that oxLDL extensively counteracts aldosterone release [16]. This finding may be related to the controversial findings of aldosterone effects on cardiovascular outcomes among HD patients. However, the effect of oxLDL on the level of circulating cortisol is not known.

Determining the effect of oxLDL on the level of serum cortisol and patient mortality in HD patients is important for the identification of candidates who are most likely to benefit from mineralocorticoid receptor blockers. Therefore, we performed an observational cohort study to evaluate whether serum cortisol affects patient mortality and whether oxLDL is an independent risk factor for elevated cortisol in maintenance HD patients.

## Methods

### Study population

Prevalent HD patients receiving HD treatment three times a week for more than 3 months at Kangnam Sacred Heart Hospital and Chuncheon Sacred Heart Hospital were enrolled in this study. Those patients who had received HD treatment for less than 3 months or with a lower frequency (<3 times/week) were excluded from the study. Patients who had been admitted to the hospital for intensive care or infection control within 3 months of study enrollment were excluded from the study because acute illness affects the level of adrenal hormones. For the same reason, patients who had been prescribed intravenous or oral corticosteroids due to organ transplantation, glomerulonephritis, dermatitis, or asthma were excluded. Patients who were taking herbal medicine or appetite stimulant within 3 months of the study date were also excluded. We further excluded patients who had cognitive dysfunction or who refused to participate in the study.

**Fig. 1:**
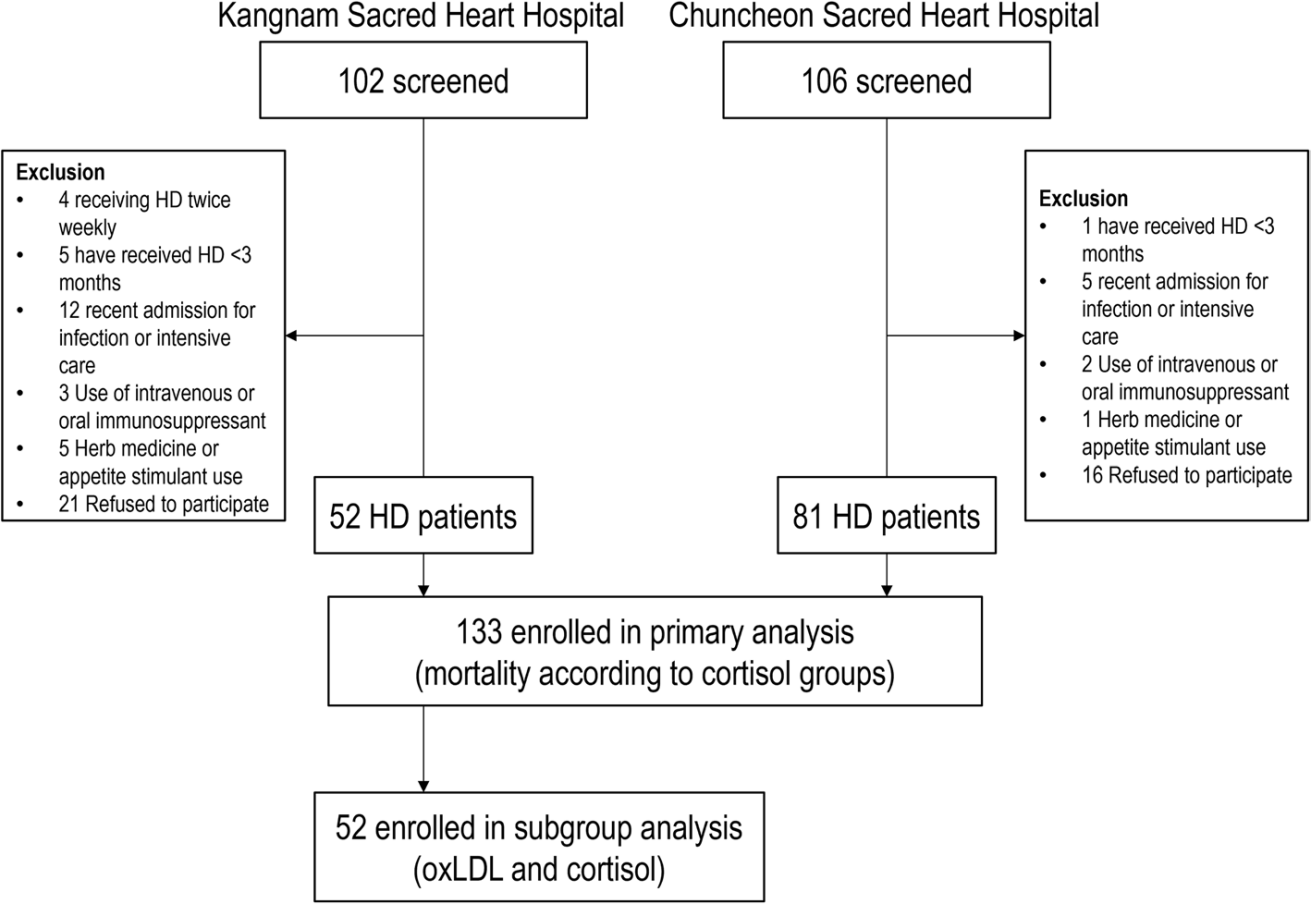
Description of study participants. A total of 208 HD patients from 2 institutions were screened, and a total of 133 participants were included in the primary analysis regarding patient mortality. Among them, 52 patients were included in the subgroup analysis to evaluate the association between oxLDL and cortisol. HD, hemodialysis; oxLDL, oxidized low-density lipoprotein

A total of 208 HD patients were screened for the study, and a total of 133 patients were enrolled. They were included in the primary mortality analysis that compared high and low cortisol groups. Among the enrolled patients, 52 patients from Kangnam Sacred Heart Hospital were included in the subgroup analysis to evaluate the effect of oxLDL on cortisol levels (Fig. 1).

This observational study was approved by the Institutional Review Board of Hallym University Kangnam Sacred Heart Hospital (IRB No. 2019-01-031) and Chuncheon Sacred Heart Hospital (IRB No. 2014-117). The patients were provided with an introduction to the study, and written informed consent was obtained from each patient before starting the study. All methods were carried out in accordance with STROBE guidelines and regulations.

### Data collection

The causes of end-stage renal disease, the presence of comorbid conditions including diabetes and hypertension, and the duration of dialysis were assessed. Interdialytic weight gain was determined using values from the 3 most recent HD treatments. Information about weekly doses of erythropoietin was collected. Laboratory evaluations were performed within 1 month of study enrollment. Plasma hemoglobin, fasting glucose, serum albumin, serum calcium and phosphorus, intact parathyroid hormone (PTH), C-reactive protein, lipid profiles, and serum sodium and serum potassium were assessed.

The echocardiogram results were collected within 3 months of study enrollment. The left ventricular ejection fraction (LVEF) was measured by M-mode transthoracic echocardiogram. The early transmitral flow velocity (E) and early mitral annular velocity (E’) were obtained by tissue Doppler imaging. Left ventricular systolic dysfunction (LVSD) was defined as LVEF less than 50% [17]. Left ventricular diastolic dysfunction (LVDD) was defined as an E/E’ ratio greater than 15 [18]. Left ventricular mass index (LVMI) was calculated as the left ventricular mass/body surface area. Left ventricular hypertrophy (LVH) was defined as an LVMI of at least 115 g/m^2^ in men and 95 g/m^2^ in women [19].

### Adrenocortical evaluation

After informed consent was obtained, baseline adrenocorticotropic hormone (ACTH) and cortisol were measured in all patients before the routine HD treatment sessions. The plasma levels of oxLDL, renin and aldosterone were measured in only 52 enrolled patients from Kangnam Sacred Heart Hospital. All patients rested for at least 10 to 15 minutes before sampling was performed. Baseline plasma and serum samples were collected in separate bottles before the routine HD treatment. Baseline blood samples were frozen at -20.0 °C until assayed. Serum cortisol was measured by immunoassay using a Cobas e 801 (Roche Diagnostics, Germany). Plasma ACTH was measured by immunoassay using an Immunulite 2000 (Siemens, UK). Plasma renin and aldosterone were measured by radioimmunoassay using a GAMMA-10 (BeckmanCoulter, Czech Republic). Plasma oxLDL was measured by enzyme-linked immunosorbent assay using a commercial kit (Mercodia AB, Sweden).

### Statistical analyses

Statistical analyses were performed using SPSS version 20.0 (IBM Corp., Armonk, NY, USA). The patients were divided into two groups according to the median cortisol and aldosterone levels. Baseline characteristics were compared between the high (baseline cortisol ≥ 10 μg/dL) and low (baseline cortisol < 10 μg/dL) cortisol groups. In addition, characteristics were compared between the high (baseline aldosterone ≥ 7.9 ng/dL) and low (baseline aldosterone <7.9 ng/dL) aldosterone groups. Continuous variables with a normal distribution are presented as the mean±standard deviation, while those with a skewed distribution are presented as the median [interquartile range]. Categorical variables are presented as frequencies. Student’s t test and the Mann–Whitney U test were performed to compare continuous variables. The chi-square test was performed to compare categorical variables between the groups. Multivariate Cox regression analysis was performed to compare overall survival between the groups after adjusting for other confounding factors. We also compared the level of oxLDL between the two cortisol groups and the two aldosterone groups. Multivariate logistic regression analysis was performed to assess oxLDL as an independent variable for the high cortisol group. A *P* value < 0.05 was considered to indicate statistical significance.

## Results

### Baseline characteristics according to baseline cortisol level

A total of 133 prevalent HD patients were enrolled from two institutions. The mean age was 62.9±10.5 years old, and the average duration of HD treatment was 5.5±5.0 years. Baseline characteristics were compared between the high and low cortisol groups (Table 1). Baseline cortisol levels in the high and low cortisol groups were 12.9±2.5 μg/dL and 7.4±1.9 μg/dL, respectively (*P*<0.001). Baseline ACTH levels were also different between the groups (41.5±22.3 pg/mL vs. 28.6±15.2 pg/mL, *P*<0.001). Compared with the low cortisol group, the high cortisol group showed a greater proportion of patients with cardiovascular disease (59.7% vs. 39.4%, *P*=0.019) and LVSD (25.9% vs. 8.0%, *P*=0.016). Meanwhile, the patients in the high cortisol group showed lower serum sodium levels than those in the low cortisol group (137.5±3.9 mg/dL vs. 139.0±3.6 mg/dL, *P*=0.024). However, the proportion of patients in the high and low cortisol groups with LVH (75.5% vs. 79.2%, respectively; *P*=0.658) or LVDD (52.0% vs. 55.1%, respectively; *P*=0.757) did not differ according to the baseline cortisol levels.

**Table 1:** Baseline characteristics according to baseline cortisol level

Variables	Total	High cortisol group (***n=***67)	Low cortisol group (***n=***66)	*P* value
Serum cortisol (μg/dL)	10.2±3.6	12.9±2.5	7.4±1.9	<0.001
ACTH (pg/mL)	31.7 [19.8, 47.1]	38.3 [23, 53.7]	24.9 [17.5, 37.8]	0.001
Age (yr)	62.9±10.5	63.4±10.1	62.4±11.0	0.594
Male (%)	65 (48.9)	31 (46.3)	34 (51.5)	0.545
HD duration (yr)	4.2 [1.8, 7.7]	4.2 [2.2, 8.3]	4.3 [1.3, 6.9]	0.439
Diabetes mellitus (%)	91 (68.4)	49 (73.1)	42 (63.6)	0.239
Hypertension (%)	127 (95.5)	65 (97.0)	62 (93.9)	0.441
Cardiovascular disease (%)	66 (49.6)	40 (59.7)	26 (39.4)	0.019
Dose of erythropoietin (IU/week)	8000 [4000, 12000]	10000 [4000, 12000]	4500 [3000, 9000]	0.008
Body mass index (kg/m^2^)	23.0±4.3	23.1±3.9	22.9±4.7	0.806
Interdialytic weight gain (kg)	2.36±0.85	2.39±0.83	2.34±0.88	0.736
Kt/V	1.68±0.3	1.62±0.28	1.68±0.35	0.348
Plasma hemoglobin (g/dL)	10.5±1.0	10.6±1.1	10.4±0.8	0.287
Glucose (mg/dL)	168.5±76.4	174.4±83.3	162.4±68.8	0.366
Serum albumin (g/dL)	3.8±0.4	3.8±0.3	3.7±0.4	0.156
Sodium (mEq/L)	138.3±3.8	137.5±3.9	139.0±3.6	0.024
Potassium (mEq/L)	4.7±0.8	4.8±0.7	4.7±0.8	0.674
Serum calcium (mg/dL)	8.9±0.8	9.0±0.7	8.7±0.9	0.134
Serum phosphorus (mg/dL)	4.4±1.4	4.5±1.3	4.3±1.6	0.429
Intact PTH (pg/mL)	279 [113.1, 484.3]	290 [143, 433.3]	257.7 [99.7, 526.7]	0.568
Total cholesterol (mg/dL)	133.7±35.4	135.1±39.6	132.4±30.8	0.664
LDL-cholesterol (mg/dL)	72.3±23.7	72.4±26.4	72.1±20.8	0.943
C-reactive protein (mg/L)	1.4 [0.7, 3.2]	1.5 [1.0, 2.9]	1.2 [0.6, 3.3]	0.311
LVH (%)	78 (77.2)	40 (75.5)	38 (79.2)	0.658
LVSD (%)	18 (13.5)	14 (25.9)	4 (8.0)	0.016
LVDD (%)	53 (39.8)	26 (52.0)	27 (55.1)	0.757

*ACTH* adrenocorticotropic hormone; *HD* hemodialysis; *Kt/V* dialysis efficiency; *LDL* low-density lipoprotein; *LVDD* left ventricular diastolic dysfunction; *LVH* left ventricular hypertrophy; *LVSD* left ventricular systolic dysfunction

### Patient survival according to cortisol groups

**Fig. 2:**
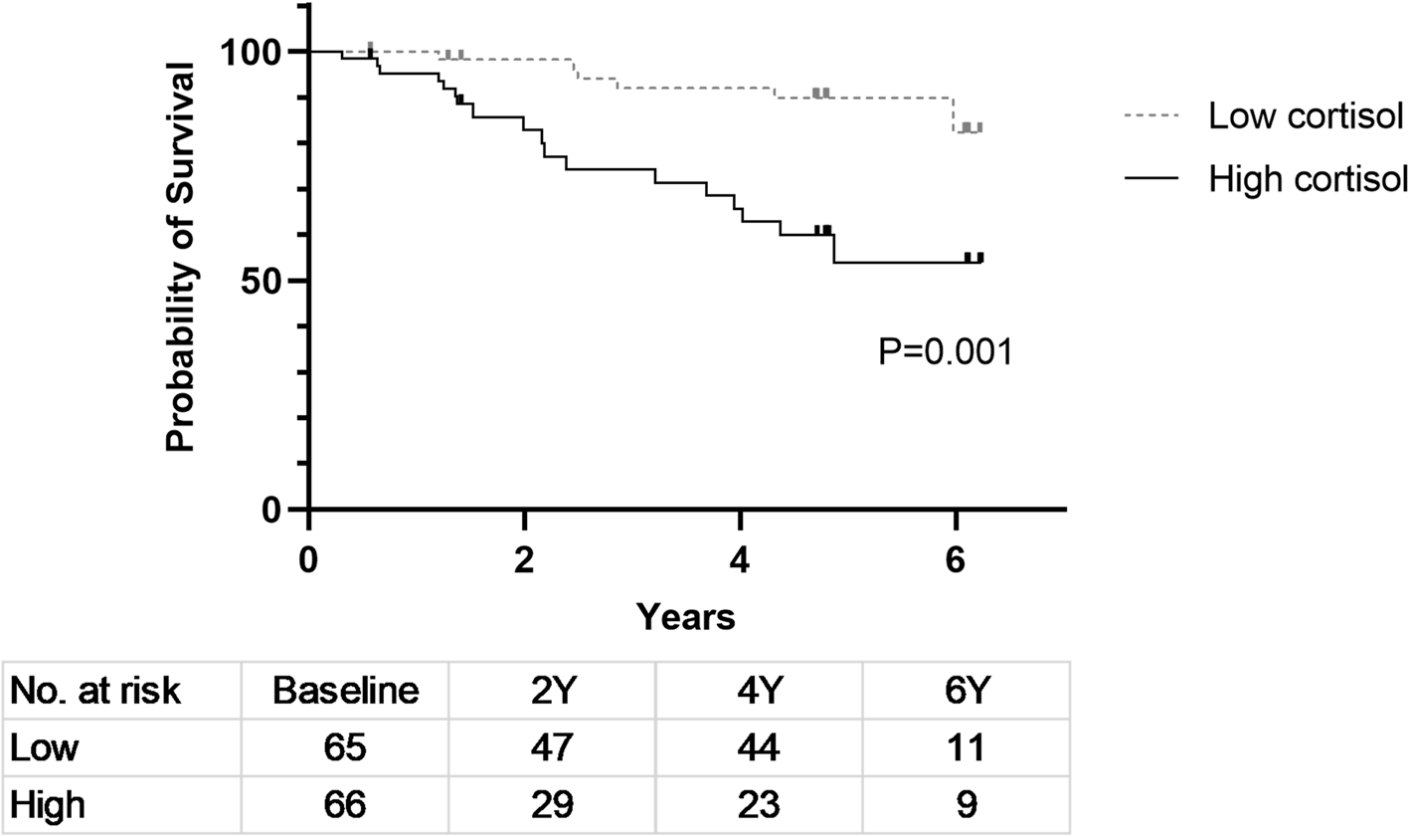
Patient survival according to cortisol groups. During a mean follow-up duration of 3.3±2.0 years, a total of 24 patients died. In the Kaplan–Meier survival analysis, the high cortisol group showed worse survival than the low cortisol group (18 deaths vs. 6 deaths, respectively; *P*=0.001). All 6 cardiovascular deaths occurred among patients in the high cortisol group

During a mean follow-up duration of 3.3±2.0 years, a total of 24 patients died. The most common causes of death were cardiovascular events (*n=*6; 25%) or septic shock (*n=*6; 25%). Other causes of death included cancer (*n=*2), hyperkalemia (*n=*1), pulmonary edema (*n=*1), and malnutrition (*n=*1). The exact causes of death were unknown in 7 cases. In the Kaplan–Meier survival analysis, the high cortisol group showed worse survival than the low cortisol group (18 deaths in the high cortisol group vs. 6 deaths in the low cortisol group, *P*=0.001; Fig. 2). All 6 cardiovascular deaths occurred among patients in the high cortisol group. The nonsurvivor group was younger than the survivor group (61.8±10.4 years vs. 68.0±9.7 years, *P*=0.009) and showed a higher proportion of diabetes mellitus (87.5% vs. 64.2%, *P*=0.026) and cardiovascular disease (87.5% vs. 41.3%, *P*<0.001) (Table 2). In addition, serum albumin was lower (3.6±0.3 vs. 3.8±0.4 g/dL, *P*=0.067) and serum potassium was higher (5.0±0.9 vs. 4.7±0.7 mEq/L, *P*=0.077) in the nonsurvivor group compared to the survivor group, with marginal statistical significance. However, among 52 patients with available plasma aldosterone level measurements, the patients with low plasma aldosterone showed poorer survival than patients with high plasma aldosterone, without statistical significance (15.4% vs. 3.8%, *P*=0.35, Supplementary Table 1). After adjusting for age, sex, the presence of diabetes mellitus, plasma hemoglobin, serum albumin, serum potassium, and LVSD, serum cortisol remained an independent risk factor for patient mortality (hazard ratio 1.234, 95% confidence interval 1.022-1.49, *P*=0.029; Table 3).

**Table 2:** Baseline characteristics according to patient survival

Variables	Nonsurvivors (***n=***24)	Survivors (***n=***109)	*P* value
Age (yr)	61.8±10.4	68.0±9.7	0.009
Male (%)	11 (45.8)	54 (49.5)	0.742
HD duration (yr)	4.7 [2.6, 9.0]	4.2 [1.6, 7.6]	0.274
Diabetes mellitus (%)	21 (87.5)	70 (64.2)	0.026
Hypertension (%)	24 (100.0)	103 (94.5)	0.591
Cardiovascular disease (%)	21 (87.5)	45 (41.3)	<0.001
Dose of erythropoietin (IU/week)	9000 [4000, 12750]	8000 [3500, 12000]	0.303
Body mass index (kg/m^2^)	22.9±2.9	23.1±4.5	0.893
Interdialytic weight gain (kg)	2.49±0.88	2.34±0.84	0.457
Kt/V	1.62±0.28	1.66±0.32	0.607
Plasma hemoglobin (g/dL)	10.5±1.2	10.5±0.9	0.783
Glucose (mg/dL)	189.1±76.7	163.9±75.9	0.145
Serum albumin (g/dL)	3.6±0.3	3.8±0.4	0.067
Sodium (mEq/L)	138.0±4.8	138.3±3.6	0.658
Potassium (mEq/L)	5.0±0.9	4.7±0.7	0.077
Serum calcium (mg/dL)	8.9±0.6	8.8±0.8	0.736
Serum phosphorus (mg/dL)	4.4±1.7	4.4±1.4	0.706
Intact PTH (pg/mL)	156.6 [52.2, 474.5]	302 [139.5, 484.3]	0.139
High cortisol group (%)	18 (75.0)	49 (45.0)	0.008
Total cholesterol (mg/dL)	128.6±35.4	134.9±35.5	0.438
LDL-cholesterol (mg/dL)	67.7±22.9	73.3±23.9	0.301
C-reactive protein (mg/L)	2.7 [1.0, 11.2]	1.2 [0.7, 2.9]	0.082
LVH (%)	11 (73.3)	67 (77.9)	0.741
LVSD (%)	5 (33.3)	13 (14.6)	0.131
LVDD (%)	9 (64.3)	44 (51.8)	0.384

*HD* hemodialysis; *Kt/V* dialysis efficiency; *LDL* low-density lipoprotein; *LVDD* left ventricular diastolic dysfunction; *LVH* left ventricular hypertrophy; *LVSD* left ventricular systolic dysfunction

**Table 3:** Multivariate Cox proportional hazard model for patient death

Variables	Hazard ratio	95% confidence interval	***P*** value
Age (per year)	1.058	0.991-1.129	0.093
Male (vs. female)	0.679	0.216-2.131	0.507
Diabetes mellitus	7.312	0.855-62.55	0.069
Plasma Hb (per 1 g/dL increase)	0.891	0.49-1.619	0.891
Serum albumin (per 1 g/dL increase)	0.528	0.077-3.633	0.517
Serum potassium (per 1 mEq/L increase)	1.198	0.609-2.357	0.6
LVSD	2.975	0.89-9.947	0.077
Serum cortisol (μg/dL)	1.234	1.022-1.49	0.029

A single model of multivariate Cox regression was used to define the independent risk factors for patient death. *CI* confidence interval; *HR* hazard ratio; *LVDD* left ventricular diastolic dysfunction; *LVSD* left ventricular systolic dysfunction

### OxLDL level as an independent risk factor for high cortisol level

**Fig. 3:**
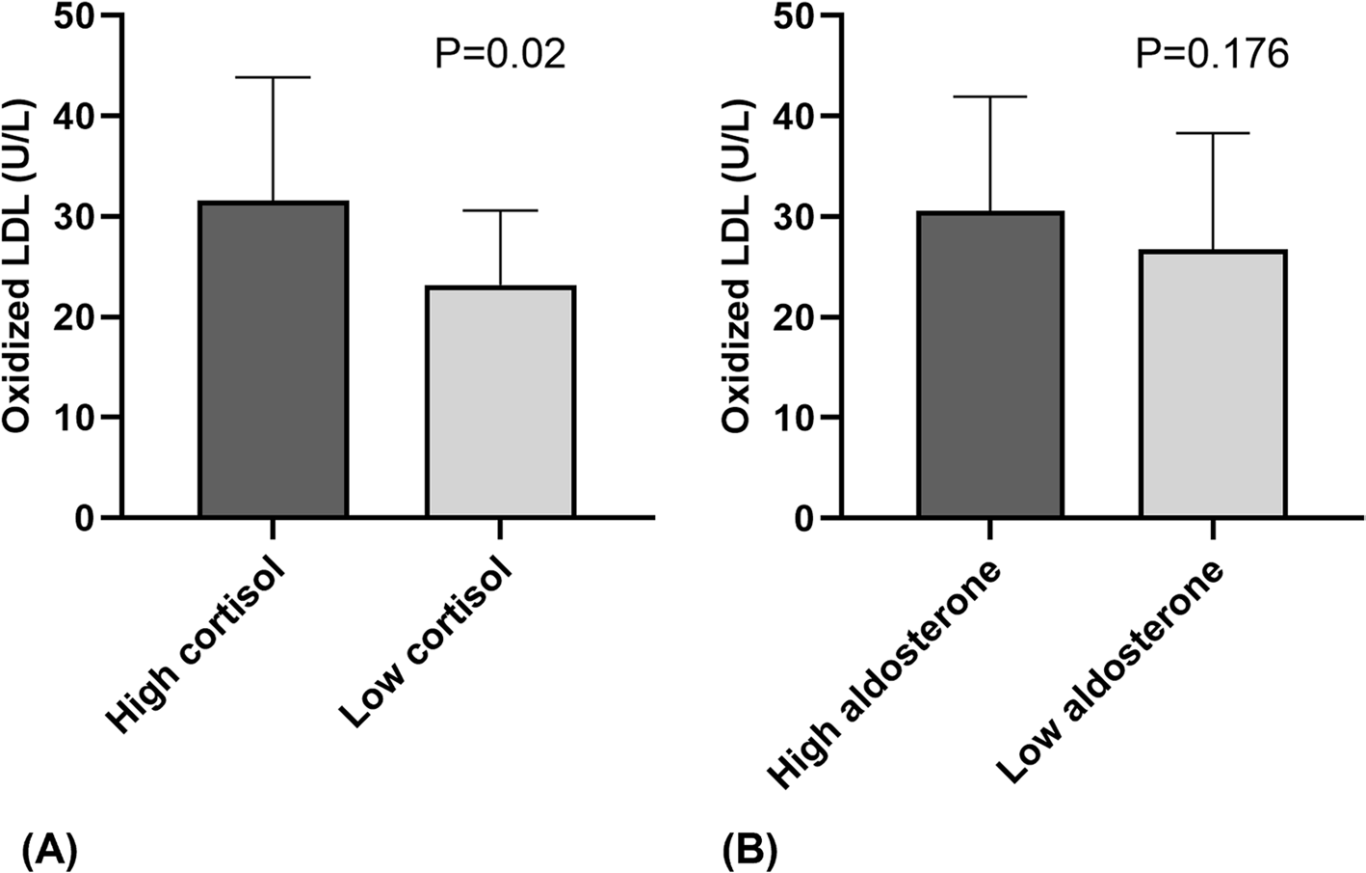
Serum oxidized low-density lipoprotein (oxLDL) levels according to adrenal hormone groups. **A** The high cortisol group showed higher oxLDL levels than the low cortisol group (31.6±12.3 U/L vs. 23.2±7.4 U/L, *P*=0.02). **B** The oxLDL level did not differ between the high and low aldosterone groups (30.6±11.4 U/L vs. 26.8±11.6 U/L, *P*=0.176)

We analyzed the relationship between plasma oxLDL and adrenal hormone levels among 52 patients with available plasma oxLDL measurements. The high cortisol group showed higher oxLDL levels than the low cortisol group (31.6±12.3 U/L vs. 23.2±7.4 U/L, *P*=0.02; Fig. 3A). However, the oxLDL level did not differ between the high and low aldosterone groups (30.6±11.4 U/L vs. 26.8±11.6 U/L, *P*=0.176; Fig. 3B). When we performed the multivariate logistic regression analysis, plasma oxLDL was an independent factor associated with the high cortisol group (Exp(B) 1.114, 95% confidence interval 1.023-1.213, *P*=0.013; Table 4) together with LVSD (Exp(B) 12.308, 95% confidence interval 1.055-143.601, *P*=0.045). Five deaths were recognized among 52 patients during follow-up. When we divided the 52 patients into two groups according to the median oxLDL, the high oxLDL group showed more frequent mortality than the low oxLDL group, but the difference was not statistically significant (15.4% vs. 3.8%, *P*=0.35; Supplementary Table 2).

**Table 4:** Serum oxLDL as an independent risk factor for high cortisol level

Variables	Exp (B)	95% CI	***P*** value
Age	0.994	0.931-1.062	0.862
Male	3.32	0.657-16.772	0.146
Diabetes mellitus	5.075	0.706-36.477	0.107
Hypertension	0.651	0.036-11.805	0.772
Cardiovascular disease	1.033	0.202-5.291	0.969
LVSD	12.308	1.055-143.601	0.045
OxLDL	1.114	1.023-1.213	0.013
Log aldosterone	1.628	0.221-12.001	0.633

A single model of multivariate logistic regression was used to define the independent risk factors for high cortisol levels. *CI* confidence interval; *LVSD* left ventricular systolic dysfunction; *oxLDL* oxidized low-density lipoprotein

## Discussion

This observational study demonstrated that a high serum cortisol level (baseline cortisol ≥ 10 μg/dL) is associated with higher cardiovascular morbidity and is an independent predictor of all-cause mortality among HD patients. In addition, oxLDL was an independent risk factor for elevated serum cortisol levels and LVSD. However, plasma aldosterone levels were not associated with serum cortisol levels.

High serum cortisol has been suggested as a risk factor for cardiovascular events and death among patients with end-stage renal disease. Drechsler et al. demonstrated in their post hoc analysis of the German Diabetes and Dialysis Study (4D Study) that the combined presence of high aldosterone (>200 pg/mL) and high cortisol (>21.1 μg/dL) levels increases the risk of all-cause death and sudden cardiac death [2]. Another study showed that serum cortisol levels were associated with inflammation and low sodium levels, and high serum cortisol among HD patients resulted in high mortality [14]. Our study also demonstrated that high serum cortisol levels result in high patient mortality. Our data also demonstrated that the high cortisol group showed lower serum sodium levels than the low cortisol group. Unfortunately, due to the small number of patients included in the analysis and some missing data, our study failed to show associations of the marker of inflammation and serum cortisol or patient death. In addition, a single measurement of C-reactive protein or serum sodium levels may not accurately reflect the status of chronic inflammation. However, our study demonstrated that serum cortisol can be a useful predictive marker for cardiovascular morbidity and all-cause mortality among HD patients under oxidative stress. Yamaji et al. also demonstrated that serum cortisol can be a useful predictive marker for cardiac events only if oxLDL was elevated in patients with chronic heart failure [13].

A previous study demonstrated that patients with end-stage renal disease have a nearly 10-fold elevation of oxLDL compared with healthy controls [20]. OxLDL may accumulate within macrophages, which may enhance chemotactic activity and result in direct injury to endothelial cells [21]. OxLDL also impairs the anti-inflammatory properties of the endothelium and enhances proinflammatory markers, including cytokines, chemokines, and growth factors [22–24]. Therefore, oxLDL may result in endothelial cell dysfunction, microvasculature atherosclerosis, and vascular calcification. Our study also showed that plasma oxLDL levels were independently associated with high cortisol levels and LVSD. Although our study did not intend to explain the underlying pathogenesis of the effect of oxLDL on cardiac outcomes, there may be a close association among oxLDL, serum cortisol, and cardiac dysfunction.

Drechsler suggested that both cortisol and aldosterone might be important risk factors for sudden cardiac death among HD patients [2], but plasma aldosterone levels did not affect patient mortality in our study. Previous studies also showed a negative correlation with cardiovascular outcomes in dialysis patients. Kohagura et al. demonstrated that hypertensive HD patients in the low aldosterone group (<22.9 ng/dL) showed a poorer survival rate than those in the high aldosterone group (≥22.9 ng/dL) (62.5% vs. 90.6%, *P*=0.003). Previous studies suggested that this paradoxical relationship might be due to the confounding effect of volume overload, inflammation, or protein energy malnutrition [9, 10]. However, in our study, the patients with high and low aldosterone levels did not show differences in interdialytic weight gain, serum albumin levels, or C-reactive protein levels (Supplementary Table 1). Instead, the paradoxical effect of aldosterone on cardiovascular outcomes may be due to the dissociation of low circulating aldosterone levels and high tissue aldosterone levels [25]. In addition, oxLDL may counteract aldosterone, resulting in low to normal plasma aldosterone levels in highly oxidative stress conditions [16]. While the plasma aldosterone level shows contradictory results, the serum cortisol level consistently predicts poor cardiovascular outcomes [13, 14]. Serum cortisol may be elevated in proinflammatory conditions and has an anti-inflammatory action against elevated oxLDL [26]. In addition, serum cortisol may be highly increased in patients with end-stage renal disease because the activity of 11βHSD2, an enzyme that converts cortisol to the inactive hormone cortisone, decreases as renal function declines [11]. Therefore, patients on dialysis may demonstrate elevated oxLDL and serum cortisol while showing normal to low aldosterone levels.

Our study has several implications. First, serum cortisol should be considered together with plasma aldosterone to monitor the effect of mineralocorticoid receptor antagonists in patients with end-stage renal disease. Since cortisol concentration may be elevated due to increased oxLDL and reduced 11βHSD2 enzyme activity in cardiac tissue, cardiac mineralocorticoid receptors may be activated mainly by cortisol [12]. Therefore, to evaluate the effect of mineralocorticoid receptor antagonists in patients with end-stage renal disease, it is reasonable to consider both hormone effects. Second, the therapeutic use of corticosteroids should be reconsidered in patients with end-stage renal disease. While an adequate dose of corticosteroids should be used when necessary, the duration and dose of steroids should be minimized for patients with a high cardiovascular risk.

There are several limitations in this study. First, we did not evaluate nonfatal cardiovascular events during the study period. Second, we only measured oxLDL and plasma aldosterone in a small number of patients. Third, the blood sampling time was different between patients. Since plasma ACTH, serum cortisol, renin, and aldosterone have diurnal variation and can be influenced by various factors, our findings can have many confounding factors. Fourth, we are unable to explain the direct effect of oxLDL on cardiovascular morbidity and all-cause mortality in this study. Finally, the causal relationship between oxLDL and serum cortisol could not be demonstrated in this study.

## Conclusions

In conclusion, serum cortisol is a useful predictive marker for all-cause death among patients receiving HD. Oxidative stress may play an important role in increasing serum cortisol levels.

## Supplementary Information

**Additional file 1.**

## Data Availability

The dataset supporting the conclusions of this article is available from the corresponding author upon reasonable request.

## Abbreviations

ACTH: Adrenocorticotropic hormone
E: Early transmitral flow velocity
E’: Early mitral annular velocity
HD: Hemodialysis
LDL: Low-density lipoprotein
LVDD: Left ventricular diastolic dysfunction
LVEF: Left ventricular ejection fraction
LVH: Left ventricular hypertrophy
LVMI: Left ventricular mass index
LVSD: Left ventricular systolic dysfunction
oxLDL: Oxidized low-density lipoprotein
11βHSD2: 11β hydroxysteroid dehydrogenase type 2
