# Supplementary material for: High cortisol levels are associated with oxidative stress and mortality in maintenance hemodialysis patients
Source: BMC Nephrol. 2022 Mar 8;23:98. doi: 10.1186/s12882-022-02722-w (PMC8903641; doi:10.1186/s12882-022-02722-w)
Supplement: Supplementary file 1 — Additional file 1. [file 12882_2022_2722_MOESM1_ESM.docx]

**Supplementary Table 1. Baseline characteristics according to baseline aldosterone**

| **Variables** | **Total** | **Low aldosterone group (n=26)** | **High aldosterone group (n=26)** | **P-value** |
| --- | --- | --- | --- | --- |
| Plasma aldosterone (ng/dL) | 7.9 [4.9, 14.4] | 5.0 [3.8, 6.6] | 14.2 [10.7, 21.2] | <0.001 |
| Renin (ng/mL/hr) | 1.2 [0.5, 2.8] | 0.9 [0.3, 3.4] | 1.4 [0.5, 2.5] | 0.399 |
| Serum cortisol (µg/dL) | 11.6±4.4 | 11.4±4.4 | 11.8±4.5 | 0.747 |
| Age (yr) | 63.1±11.3 | 64.3±11.8 | 62.0±10.9 | 0.475 |
| Male (%) | 24 (46.2) | 12 (46.2) | 12 (46.2) | >0.99 |
| HD duration (yr) | 5.0 [2.6, 8.1] | 4.4 [2.5, 8.5] | 5.5 [2.6, 8.2] | 0.763 |
| Diabetes mellitus (%) | 37 (71.2) | 18 (69.2) | 19 (73.1) | 0.76 |
| Hypertension (%) | 49 (94.2) | 25 (96.2) | 24 (92.3) | >0.99 |
| Cardiovascular disease (%) | 23 (44.2) | 9 (34.6) | 14 (53.8) | 0.163 |
| Dose of erythropoietin (IU/week) | 8000 [4000, 12000] | 8000 [4000, 12000] | 8000 [4000, 12000] | 0.516 |
| Body mass index (kg/m^2^) | 24.4±3.9 | 24.6±4.5 | 24.2±3.1 | 0.725 |
| Interdialytic weight gain (kg) | 2.36±0.87 | 2.29±0.88 | 2.44±0.87 | 0.53 |
| Kt/V | 1.68±0.3 | 1.66±0.3 | 1.77±0.3 | 0.759 |
| Plasma hemoglobin (g/dL) | 10.7±1.0 | 10.5±1.0 | 10.9±1.0 | 0.158 |
| Glucose (mg/dL) | 186.3±84.0 | 184.4±77.2 | 188.1±91.7 | 0.876 |
| Serum albumin (mg/dL) | 3.9±0.3 | 3.8±0.3 | 3.9±0.3 | 0.926 |
| Sodium (mEq/L) | 136.5±3.5 | 136.0±4.0 | 137.0±2.9 | 0.304 |
| Potassium (mEq/L) | 4.5±0.6 | 4.5±0.5 | 4.6±0.6 | 0.47 |
| Serum calcium (mg/dL) | 9.0±0.6 | 9.1±0.5 | 9.0±0.8 | 0.287 |
| Serum phosphorus (mg/dL) | 4.7±0.9 | 4.7±1.0 | 4.8±0.8 | 0.7 |
| Intact PTH (pg/mL) | 276 [103.8, 415] | 238.5 [95.2, 377.5] | 310.5 [133.8, 441.5] | 0.442 |
| Total cholesterol (mg/dL) | 128.9±38.1 | 124.5±42.2 | 133.4±33.8 | 0.406 |
| LDL-cholesterol (mg/dL) | 74.6±25.9 | 71.2±26.8 | 78.0±25.0 | 0.348 |
| OxLDL (U/L) | 28.7±11.5 | 26.8±11.6 | 30.6±10.0 | 0.235 |
| C-reactive protein (mg/L) | 1.4 [1.0, 2.6] | 1.3 [0.9, 2.2] | 1.9 [1.0, 3.3] | 0.431 |
| LVH (%) | 46 (90.2) | 25 (96.2) | 21 (84.0) | 0.191 |
| LVSD (%) | 12 (23.5) | 7 (26.9) | 5 (20.0) | 0.56 |
| LVDD (%) | 29 (63.0) | 16 (66.7) | 13 (59.1) | 0.595 |
| Death | 5 (9.6) | 4 (15.4) | 1 (3.8) | 0.35 |

HD, hemodialysis; Kt/V, dialysis efficiency; LDL, low density lipoprotein; LVDD, left ventricular diastolic dysfunction; LVH, left ventricular hypertrophy; LVSD, left ventricular systolic dysfunction; oxLDL, oxidized-low density lipoprotein

**Supplementary Table 2.** **Clinical characteristics according to baseline oxLDL**

| **Variables** | **Total** | **Low oxLDL (n=26)** | **High oxLDL (n=26)** | **P-value** |
| --- | --- | --- | --- | --- |
| OxLDL (U/L) | 28.7±11.5 | 23.5±8.1 | 33.9±12.2 | 0.001 |
| Age (yr) | 63.1±11.3 | 64.7±11.8 | 61.5±10.8 | 0.313 |
| Male (%) | 24 (46.2) | 11 (42.3) | 13 (50.0) | 0.578 |
| HD duration (yr) | 5.7±3.8 | 5.6±3.7 | 5.7±4.0 | 0.936 |
| Diabetes mellitus (%) | 37 (71.2) | 19 (73.1) | 18 (69.2) | 0.76 |
| Hypertension (%) | 49 (94.2) | 23 (88.5) | 26 (100.0) | 0.235 |
| Cardiovascular disease (%) | 19 (36.5) | 9 (34.6) | 10 (38.5) | 0.773 |
| Body mass index (kg/m^2^) | 24.4±3.9 | 24.1±3.7 | 24.7±4.1 | 0.565 |
| Interdialytic weight gain (kg) | 2.36±0.87 | 2.31±0.97 | 2.42±0.78 | 0.66 |
| Kt/V | 1.68±0.3 | 1.72±0.3 | 1.63±0.3 | 0.266 |
| Plasma hemoglobin (g/dL) | 10.7±1.0 | 10.6±1.2 | 10.8±0.8 | 0.432 |
| Glucose (mg/dL) | 186.3±84.0 | 194.4±82.8 | 178.2±86.0 | 0.491 |
| Serum albumin (mg/dL) | 3.9±0.3 | 3.8±0.3 | 3.9±0.3 | 0.038 |
| Sodium (mEq/L) | 136.5±3.5 | 136.6±4.1 | 136.5±2.8 | 0.875 |
| Potassium (mEq/L) | 4.5±0.6 | 4.5±0.5 | 4.5±0.6 | 0.785 |
| Serum cortisol (ug/dL) | 11.6±4.4 | 10.2±4.9 | 12.9±3.6 | 0.029 |
| Total cholesterol (mg/dL) | 128.9±38.1 | 125.2±35.6 | 132.6±40.8 | 0.492 |
| LDL-cholesterol (mg/dL) | 74.6±25.9 | 75.4±23.3 | 73.7±28.7 | 0.82 |
| C-reactive protein (mg/L) | 3.42±8.1 | 2.71±3.5 | 4.13±11.0 | 0.541 |
| LVH (%) | 42 (82.4) | 22 (84.6) | 20 (80.0) | 0.726 |
| LVSD (%) | 12 (23.5) | 4 (15.4) | 8 (32.0) | 0.162 |
| LVDD (%) | 29 (63.0) | 15 (62.5) | 14 (63.6) | 0.936 |
| Death (%) | 5 (9.6%) | 1 (3.8) | 4 (15.4) | 0.35 |

HD, hemodialysis; Kt/V, dialysis efficiency; LDL, low density lipoprotein; LVDD, left ventricular diastolic dysfunction; LVH, left ventricular hypertrophy; LVSD, left ventricular systolic dysfunction; oxLDL, oxidized-low density lipoprotein
